# The Transcriptional Network in the Arabidopsis Circadian Clock System

**DOI:** 10.3390/genes11111284

**Published:** 2020-10-29

**Authors:** Norihito Nakamichi

**Affiliations:** 1 Institute of Transformative Bio-Molecules, Nagoya University, Furo-cho, Chikusa, Nagoya 464-8602, Japan; nnakamichi@itbm.nagoya-u.ac.jp; 2 Division of Biological Science, Graduate School of Science, Nagoya University, Furo-cho, Chikusa, Nagoya 464-8602, Japan

**Keywords:** circadian clock, plant development, arabidopsis, transcriptional network, transcription factors

## Abstract

The circadian clock is the biological timekeeping system that governs the approximately 24-h rhythms of genetic, metabolic, physiological and behavioral processes in most organisms. This oscillation allows organisms to anticipate and adapt to day–night changes in the environment. Molecular studies have indicated that a transcription–translation feedback loop (TTFL), consisting of transcriptional repressors and activators, is essential for clock function in *Arabidopsis thaliana* (Arabidopsis). Omics studies using next-generation sequencers have further revealed that transcription factors in the TTFL directly regulate key genes implicated in clock-output pathways. In this review, the target genes of the Arabidopsis clock-associated transcription factors are summarized. The Arabidopsis clock transcriptional network is partly conserved among angiosperms. In addition, the clock-dependent transcriptional network structure is discussed in the context of plant behaviors for adapting to day–night cycles.

## 1. Introduction

Circadian regulation of biological processes is thought to be crucial for prediction and adaptation to day–night cycles. Many metabolic, physiological and behavioral processes are under the control of the circadian clock in plants [1,2]. Leaf movement has been recognized for a long time as a marker for circadian rhythms [3]. The circadian movement of legume leaves is driven by a circadian change in turgor pressure. Although *Arabidopsis thaliana* (Arabidopsis) also shows circadian cotyledon and leaf movement, the movement is driven by differential growth of the adaxial and abaxial sides of leaves, not by turgor pressure as in the case of legumes [3,4]. Arabidopsis also has additional rhythmic movements that occur during hypocotyl elongation and flowering stem elongation [3]. The speed of circumnutation of the flowering stem is also controlled by the circadian clock [5], as is stomatal opening and closure [6] and the optimal timing for defense responses [7,8]. Important for plant metabolism, the pace of starch breakdown during the night is controlled by the circadian clock [9,10]. These phenomena coordinate to give fitness (or an adaptive advantage) to plants during 24 h day–night cycles [11,12,13]. Field experiments further indicate that the clock is crucial for plant size, branching, flowering time and fitness in Arabidopsis [14,15].

Transcriptome analyses of Arabidopsis grown under diel and circadian conditions revealed that 6 to 26% of all genes are expressed cyclically, depending on the conditions of the experiment [16,17,18]. Meta-analysis of these transcriptome analyses showed that expression of about 90% of genes is cyclic at least in one diel or under circadian conditions [19]. By mining these transcriptome data, some biological processes under clock control were found. Expression of genes for the phenylpropanoid synthetic pathway is cyclic with a peak around dawn; this timing may enable Arabidopsis to produce phenylpropanoid-related secondary metabolites, some of which absorb light to protect cells like a sunscreen [16]. The expression of auxin-signaling genes is significantly circadian regulated. In addition, auxin sensitivity is higher during late night and lower in the daytime, showing that auxin sensitivity is gated by the clock [20]. Expression of *dehydration-responsive element B1/C-repeat-binding factor* (*DREB1/CBF*) genes, encoding key transcription factors for cold-stress responses, are circadian regulated [16]. Cold-stress-induced *DREB1/CBF* expression is gated by the clock, and induction of these genes is maximal in the morning and minimal in the evening [16,21]. The gate effect on *DREB1/CBF* is disrupted in clock mutants [21,22,23]. Collectively, transcriptome analyses have revealed the identity of genes and biological processes under clock control.

Many clock-associated genes encode proteins related to transcription, and these genes constitute a transcription–translation feedback loop (TTFL) for clock function [24,25]. CIRCADIAN CLOCK-ASSOCIATED 1 (CCA1) and LATE ELONGATED HYPOCOTYL (LHY) are the closest single Myb transcription factors expressed around dawn. CCA1 and LHY repress the transcription of clock-associated genes expressed from morning to evening. Target genes of CCA1 and LHY are *EARLY FLOWERING 4* (*ELF4*), *LUXARRHYTHMO* (*LUX*), *PSEUDO-RESPONSE REGULATOR 9* (*PRR9*), *PRR7*, *PRR5*, *TIMING OF CAB EXPRESSION 1* (*TOC1*, also called *PRR1*), *COLD-REGULATED GENE 27* (*COR27*), *COR28* and *GIGANTEA* (*GI*) [26,27,28,29,30]. PRR9, PRR7 and PRR5 are transcription repressors and repress the transcription of *CCA1*, *LHY*, *REVEILLE 8* (*RVE8*), *NIGHT LIGHT-INDUCIBLE AND CLOCK-REGULATED 1* (*LNK1*), *LNK2*, *LNK3*, *LNK4* and *PRR* genes expressed during earlier phases [31,32,33]. TOC1 also represses the target genes of other PRR [34,35]. In addition, TOC1 represses *LUX*, *ELF4* and *GI* expression [35]. LUX, ELF4 and ELF3 form the protein complex known as the Evening Complex (EC) to repress *PRR9*, *PRR7* and *LUX* [36,37,38]. LNK proteins interact with RVE8, and the LNK-RVE8 complex activates expression of *PRR5* and *TOC1* [39]. COR27 and COR28 repress *PRR5* and *TOC1* expression, although COR27 and COR28 have no DNA-binding activity in vitro. Thus, it is likely that COR27 and COR28 associate with *TOC1* and *PRR5* promoters by interacting with other transcription factors [40]. LIGHT-REGULATED 1 (LWD1) and LWD2 bind to TEOSINTE BRANCHED 1-CYCLOIDEA-PCF20 (TCP20) and TCP22 on the *CCA1* promoter region and activate *CCA1* transcription [41]. TCP21, known as CCA1-HIKING EXPEDITION (CHE), represses *CCA1* [42]. This highly wired genetic network in which the expression of clock-associated genes is modulated by internal and external signals increases the variation in expression timing, a feature that may contribute to the ability of plants to adapt to environmental changes that originate from day–night cycles [10,43,44,45].

## 2. Transcriptional Networks under Clock Regulation

### 2.1. The Transcriptional Network of CCA1 and LHY

Genetic studies have indicated that *CCA1* and *LHY* are essential for the clock and influence output biological processes such as hypocotyl elongation, flowering time, cold-stress responses and photosynthesis [11,46,47,48,49]. A chromatin immunoprecipitation and deep sequencing (ChIPseq) study demonstrated that CCA1 associates with more than 1000 genomic loci in the Wassilewskija (WS) accession under constant light or diel conditions [27]. Another ChIPseq study indicated that CCA1 associates with about 400 loci in the Col-0 accession [26]. Gene numbers may be different in these two studies because the statistical values for finding peaks of short reads were different, and different materials were used (antibodies and Arabidopsis accessions). Comparison of these two studies revealed at least 254 loci of CCA1 occupancy in the Arabidopsis genome. The evening element (EE, AAATATCT) is enriched in the CCA1-immunoprecipitated DNA sequences [26,27], a result that is compatible with the finding that CCA1 binds to EE in vitro [16,50]. Other DNA sequences were also enriched in the CCA1-immunoprecipitated DNA, possibly suggesting that CCA1 associates with the target genes by constructing complexes with other transcription factors in vivo [26,27].

**Figure 1:**
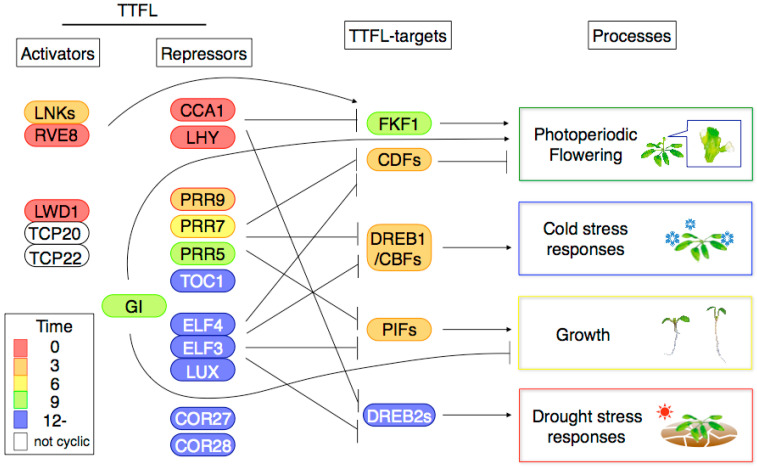
A model of the transcriptional network of the Arabidopsis circadian clock. The clock transcription–translation feedback loop (TTFL) regulates biological processes through regulating key genes (TTFL-targets). Interactions within the clock TTFL are not shown. ‘Time’ is the peak time for mRNA expression under 12 h light/12 h dark conditions in DIURNAL [105]. Other TTFL-targets are detailed in the text.

Many CCA1-bound genes are expressed in the evening (Figure 1). In the *cca1 lhy* double mutant, expression peaks of CCA1-target genes were advanced to morning phases [26,48]. Thus, CCA1 can determine the phase for expressing target genes [26]. The ChIP study also indicated that some CCA1-targets are not expressed in a diel or circadian rhythmic manner [27]. This result may suggest that there are genes whose transcription is regulated by CCA1 in specific conditions or in specific tissues or cells so that the effect of CCA1 is not clear under all growth conditions tested.

A study combining ChIPseq of LHY and transcriptome analysis identified the target genes of LHY [29]. Comparison between CCA1-binding loci and LHY-binding loci indicates that these two transcription factors have highly shared targets. However, there are ABA-related genes that are represented only in the LHY-target genes. Expression of *NINE-CIS-EPOXYCAROTENOID DIOXYGENASE 3*, encoding a rate-liming enzyme for abscisic acid (ABA) biosynthesis, was strongly reduced in transgenic plants overexpressing *LHY*. Other genes for ABA-signaling components were altered in *lhy* loss-of-function and *LHY* over-expressing constructs. ABA sensitivity is also changed in these lines, showing ABA signaling control by *LHY* [29].

### 2.2. The Transcriptional Network of LNK1 and RVE8

Given that *lnk1 lnk2* double mutants and *rve8 rev6 rev4* triple mutants had long periods and altered clock-output processes, these genes are necessary for clock function [51,52,53,54,55]. A recent study indicates that RVE8 binds to LNK proteins, and this complex bound to the *PRR5* and *TOC1* promoters recruits the basal transcriptional machinery to induce *PRR5* and *TOC1* expression [39]. Genome-wide gene expression analysis indicated that *LNK1* and *LNK2* activate genes whose expression occurs in the afternoon [52]. These gene sets include *FLAVIN-BINDING, KELCH REPEAT* and *F BOX 1* (*FKF1*), a key regulator of flowering time [56,57], suggesting that *LNK* genes control flowering through *FKF1* expression (Figure 1). Transcriptome analysis using transgenic plants expressing chemically induced RVE8 identified the primary target genes of RVE8 [51]. RVE8-target genes are expressed in the evening and include genes involved in responses such as external stimuli, defense and temperature changes [51].

### 2.3. The Transcriptional Network of PRR9, PRR7, PRR5 and TOC1

PRR9, PRR7, PRR5 and TOC1 (PRR1) proteins are sequentially expressed from early morning to midnight [58,59,60] and are crucial for clock function. Altered phenotypes of clock-output processes such as flowering time, hypocotyl elongation, cold-stress responses, drought-stress responses, greening, and metabolite alteration in *prr* and *toc1* mutants indicated the importance of *PRR* and *TOC1* for these biological processes [22,61,62,63,64,65,66,67,68,69]. Transcriptome analyses of *prr* mutants and transgenic lines and ChIPseq studies of PRR9, PRR7 and PRR5 proteins indicated that the three PRR proteins share target genes [31,32,33] (Figure 1). The target genes are significantly enriched in genes encoding transcription factors. The three PRR proteins directly repress expression of *CYCLING DOF FACTOR* (*CDF*) genes that encode transcription factors capable of repressing the florigen gene *FT* and *CONSTANS* (*CO*), a transcriptional activator of *FT* [70]. PRR9, PRR7, PRR5 and TOC1 repress *PHYTOCHROME INTERACTING FACTOR 4* (*PIF4*) and *PIF5,* two genes that encode transcription factors capable of inducing hypocotyl growth in the dark [71,72]. The three PRR proteins repress *DREB1/CBF* genes that encode transcription factors for cold-stress responses [73]. Except for the *DREB1* gene, PRR-target genes tend to have expression patterns with peaks at dawn and the early morning. The gate effect of cold stress-dependent *DREB1* induction was highly attenuated in the *prr9 prr7 prr5* triple mutants [22]. Expression of these PRR-target genes was upregulated in the *prr9 prr7 prr5* mutants compared to wild type, suggesting that these PRR proteins repress the target genes [31]. PRR9, PRR7 and PRR5 possess a transcriptional repression motif [59] that recruits histone deacetyl transferases to the PRR-targets *CCA1* and *LHY* [74].

Recombinant CCT domains of PRR proteins, except PRR3, bind to a T1ME DNA sequence (TGTG or CACA) of the *CCA1* promoter in vitro [34]. A recent study further demonstrated that the CCT domain, NF-YB, and NF-YC form a complex, and the complex binds to CCACA elements in vitro [75]. ChIPseq studies showed that the G-box (CACGTG)-like DNA sequences were enriched in the PRR-immunoprecipitated fractions [31,32,33,35], though it is possible that the PRR proteins associate with G-box like elements through protein–protein interaction due to cross-linking during the ChIP procedure. Not all PRR-bound DNA contains CCACA- or G-box-like sequences, suggesting that PRRs may associate with DNA by binding to other transcription factors in vivo [31,33]. Indeed, PRR proteins bind to some transcription factors. PRR, PIF and CO are known to interact with PRRs [76,77,78,79,80,81]. PRRs can modulate the stability or activity of these proteins. Given that PRRs regulate transcription of *PRR*, *PIF* and *CO* directly or indirectly [31,82], it is possible to propose that PRRs regulate these transcription factors at the transcriptional and post-translational steps. Regulation at multiple steps may allow these transcription factor activities to be under strict and precise control by the clock.

### 2.4. The Transcriptional Network of ELF3, ELF4 and LUX

The EC, an essential protein complex in the clock, consists of ELF3, ELF4 and LUX [38]. The *elf3*, *elf4* and *lux* mutants impair clock output processes such as flowering time regulation and hypocotyl elongation [36,37,83,84,85,86,87,88,89]. ChIPseq of ELF3, ELF4 and LUX confirms that these proteins share target genes [90] (Figure 1). Enriched DNA sequences found in and bound by LUX are the LBS and the G-box; the former is bound by LUX in vitro [37], whereas the latter is bound by b-HLH and b-ZIP transcription factors. LUX may bind to such G-box-binding transcription factors on G-boxes. Most of the EC-target genes are upregulated in the *elf3*, *elf4* and *lux* mutants, suggesting a repressive role of the EC [90]. The EC interacts with the SWI2/SNF2-RELATED (SWR1) complex to regulate deposition of H2A.Z-nucleosomes at the EC-targets [91].

An intriguing feature of EC function is its functioning at cooler conditions, which explains part of the clock’s entrainment mechanism that uses temperature changes as environmental time cues. Genome-wide gene expression changes resulting from the *elf3* mutation compared to the wild-type at 22 °C is correlated to that at 27 °C compared to 22 °C in the wild-type [90]. The EC-target genes, such as *PRR7*, *PRR9*, *LUX* and *PIF4*, are induced by a warm-temperature shift during the early night; this induction is diminished in the mutants whose EC components are impaired [92,93]. In vitro experiments further show that the EC binds to the LBS with high affinity under cooler conditions [94]. In addition to the temperature-dependent response at the molecular level, the EC regulates temperature responses beyond organs, given that ELF4 translated in shoot tissues moves to roots in cooler temperatures and sets the clock in root tissues [95].

The EC targets two key light signaling genes, *PIF4* and *PIF5* [38]. ChIPseq revealed that the EC targets additional light-signaling genes whose expression is rapidly induced by light [93]. The EC also targets genes implicated in the heat- and cold-stress responses, *DREB1/CBF* and *DREB2*. Other prominent biological processes of the EC-targets include growth-related processes. For example, *BANQUO1* (*BNQ1*)/ *PACLOBUTAZOL1 RESISTANCE1* (*PRE1*) and *BNQ2/PRE2*, implicated in cell elongation and flowering time, are directly regulated by the EC [90]. The EC targets *ARABIDOPSIS RESPONSE REGULATOR6* (*ARR6*), *ARR7*, *CYTOKININ RESPONSE FACTOR* (*CRF4*) and *CRF5* genes implicated in cytokinin responses. Collectively, the EC directs key genes involved in photosynthesis, temperature stress and growth [90].

### 2.5. The Transcriptional Network of GI

Given that null mutants of *GI* alter the period length and amplitude, *GI* is required for clock function [96]. Despite its essential role, the molecular function or biochemical activity of GI remained unknown for a long time. Recent biochemical and molecular biology studies have suggested that GI possesses multifunctional biochemical activities. GI possesses protein chaperone activity and helps ZEITLUPE (ZTL) mature into an active form [97]. Since ZTL is the ubiquitin E3-ligase for TOC1 and PRR5 [60,98,99], GI affects the levels of these proteins [97]. Given that the *gi* and *ztl* mutants have shorter and longer periods, respectively, *GI* seems to control period length through ZTL-independent pathways. Recently, a chromatin immunoprecipitation assay showed that GI protein associates with the *CCA1* promoter [100]. GI does not have a typical DNA-binding domain, suggesting that other proteins bridge between GI and the *CCA1* promoter. In addition, GI binds to PIF transcription factors and modulates the stability and activity of PIFs [100]. PIFs bind to the *CCA1* promoter in the light–input pathway and affect *CCA1* transcription [100]. Thus, GI controls *CCA1* transcription in multiple ways, including controlling the stability of TOC1 and PRR5, two transcriptional repressors of *CCA1*.

The ChIPseq study indicated that genomic loci bound by GI and PIF3 overlap significantly, showing close interaction between these proteins [100] (Figure 1). At the highest GI-binding signal loci (top 10%), PIF3-binding signals were greater in the *gi* null mutants, suggesting that GI inhibits PIF3 binding to DNA. Common targets of PIF3 and GI are enriched in genes related to the circadian clock, the response to water deprivation, the response to chitin, the ethylene-activated signaling pathway and transcription. Although the impact of GI-PIF interaction on the overall physiology of plants still needs to be determined, the interaction is crucial for light input to the clock and for controlling hypocotyl elongation through regulating *CCA1* and *PIF3-LIKE1* genes [100].

*GI* is known to regulate not only the clock and hypocotyl elongation but also a wide range of physiological processes [101]. Photoperiodic flowering time regulation is one *GI*-controlled developmental process in which *GI* functions in at least three pathways (Figure 1). First, GI binds to and modulates the activity of FKF1, a ubiquitin E3 ligase that targets the degradation of flowering repressor transcription factors, CDF proteins that inhibit the transcription of *FT* and *CO* genes [56,57]. Simultaneously, GI associates with *FT* promoter regions [102]. *GI* also upregulates miR172 that targets transcriptional repressors of *FT* [103]. GI interacts with SPINDLY protein, an O-linked beta-N-acetylglucosamine transferase that modulates flowering time, thus confirming an additional pathway in which *GI* controls flowering [104].

### 2.6. Possible Interactions among Clock Transcription Factors for Regulating Gene Expression

There are some common target genes that are regulated by different classes of clock-transcription factors (Figure 1). *PIF4*, *PIF5*, three *DREB1/CBF* genes, *CDF2*, *CDF3*, *RVE1*, *RVE7*, *B-BOX DOMAIN PROTEIN 24* (*BBX24*) and *EARLY LIGHT-INDUCABLE PROTEIN 1* (*ELIP1*) genes are targeted by PRR5 and the EC. *DREB2C*, *DREB2H*, *RVE7*, *GI*, *PRR7*, *PRR9* and *LUX* genes are targeted by CCA1 and the EC. *RVE7*, *PRR7* and *PRR9* are targets of PRR5 and CCA1. The combinatorial binding of PRR5 and CCA1 may cause shifts in the timing of gene expression of the target genes [26]. How different classes of clock-associated transcription factors, including RVE, LNK and TCP proteins, interact to regulate target output genes is an intriguing and yet to be determined question.

## 3. An Evolutionary View of Clock-Dependent Transcriptional Networks in Plants

### 3.1. The Transcriptional Network under Clock Control in Angiosperms

Details about the molecular components of the plant clock have been examined thoroughly in Arabidopsis; however, transcriptome analyses of other plants have indicated that the clock transcriptional network is partly conserved among angiosperms [106]. Comparable diel transcriptome analyses of Arabidopsis, Populus and rice showed that the phases of expression peaks of many circadian clock-associated genes, such as *CCA1/LHY*, *GI*, *LUX*, *PRR* and *TOC1*, are highly similar among these plants [106]. Orthologous genes of the clock-output pathways are expressed at a similar time-of-day among these plants [106]. Furthermore, cis-regulatory elements found in the Arabidopsis clock transcriptional network, such as morning element (ME), the G-box for morning-phased expression, EE, GATA for evening expression and the protein box (PBX)/ the telo-box (TBX)/ the starch box (SBX) for midnight expression, are conserved in rice and Populus [19,106]. Some homologs of the Arabidopsis PRR5-target genes in rice and Populus are repressed when *PRR* homologs are expressed, suggesting that the Arabidopsis PRR5 transcriptional network is partly conserved in rice and Populus [107].

Output biological processes under clock control are divergent among species, organs, tissues and cells. Recently, the expression of an outward anion channel gene was reported to peak around dawn in flexor motor cells, but not in extensor motor cells, in the pulvinus of Samanea saman, a mimosoid tree, suggesting that anion channel-dependent cell shrinkage of flexor cells during the daytime is crucial for leaf movement [108]. Solar tracking of sunflower stems is driven by opposing growth rhythms on the east and west sides of the stems [109]. Sunflower *LHY*- and *TOC1*-homolog genes are expressed at a specific time-of-day similar to those in Arabidopsis. The expression of these genes is similar in both sides of sunflower stems; however, two homologs of genes involved in phototropism are expressed differently on the opposite sides, suggesting the molecular basis underpinning solar tracking [109]. In Arabidopsis, the clock in epidermal cells controls *PIF4* expression to coordinate thermo-responsive growth [110,111]. Photoperiodic flowering is regulated by the vascular clock in Arabidopsis [112]. These studies clearly indicate the importance of tissue or cell-specific clock-output gene regulation, which is also indicated by time-course transcriptome analyses of distinct organs or tissues in Arabidopsis [111,112,113]. In addition, clock parameters such as the period and amplitude are different among cells in whole plants [114,115], though there are couplings of clocks among cells [113,116]. In the future, single-cell level transcriptome analyses will identify how cell-type-specific clock output is controlled.

In rice, *OsCCA1* and *OsTOC1* control tillering and panicle development by regulating genes for strigolactone signaling [75]. *OsGI* is essential for robust diel transcriptome rhythms in the field [117]. Recent molecular–genetic studies have suggested that flowering times of some crops were optimized by naturally or artificially occurring mutations in the orthologues of Arabidopsis clock-associated genes [118,119,120]. This evidence strongly suggests that the core clock network described in Arabidopsis is conserved in angiosperms, but divergent means of regulating clock outputs are consequences of distinct strategies for adapting to the environment.

### 3.2. The Transcriptional Network under Clock Control in the Plant Lineage

Comparison of genomes and diel transcriptome data for organisms spanning the Archaeplastida provides some evolutionary insight into diel gene expression in the plant lineage [121,122]. These studies indicate that homologs of the clock-associated genes in Arabidopsis are conserved among tracheophytes (from ferns to flowering plants). Homologs of *CCA1/LHY*, *RVE* and *LUX* are conserved in Archaeplastida, whereas other clock-associated genes are not. A *GI* homolog is not found in the bryophyte *Physcomitrella patens* [121], but *GI* and *FKF1* homologs are crucial for day-length-dependent growth-phase transition in another bryophyte, *Marchantia polymorpha* [123]. *PRR*, *TOC1*, *ELF3*, *ELF4* and *GI* homologs are not found in *Cyanophora paradoxa* (early-diverging alga, glaucophyte) or in *Porphyridium purpureum* (rhodophyte). *PRR* and *TOC1* are found in two species of chlorophyte, *Chlamydomonas reinhardtii* and *Ostreococcus tauri* [121,124]. Ostreococcus CCA1 binds to EE on the Ostreococcus *TOC1* promoter, and this binding is required for evening-phased gene expression [124], showing a similar relationship between CCA1 and *TOC1* in Arabidopsis. Two *LUX* homologs are required for Chlamydomonas clock function [125], though homologs of *ELF4* and *ELF3* are absent in this alga. A *LUX* homolog (*ROC75*) is expressed during the day, and ROC75 protein directly represses a homolog of *CCA1/LHY*. These results indicate that the TTFL for clock function differs between Chlamydomonas and Arabidopsis [125].

## 4. Conclusions

Thanks to intrinsic genomic approaches such as RNAseq and ChIPseq using Arabidopsis seedlings, the target genes of CCA1/LHY, LNKs, RVE8, PRRs/TOC1, the EC and GI have been characterized. The network architecture provides time-of-day information from the clock TTFL to output pathways. Comparative transcriptome analyses indicate that the clock TTFL is conserved in many land plants; however, clock output gene expression is thought to be organ-, tissue- or cell-specific in order to control the output properly to adjust to environmental fluctuations coming from day–night or seasonal changes. This illustration of the transcriptional network under clock control at the organ or tissue level in diverse plants provides deeper insight into the ability of plants to adapt to 24 h and seasonal cycles.
